# Polymeric Micelles in Cancer Immunotherapy

**DOI:** 10.3390/molecules26051220

**Published:** 2021-02-25

**Authors:** Zhuoya Wan, Ruohui Zheng, Pearl Moharil, Yuzhe Liu, Jing Chen, Runzi Sun, Xu Song, Qiang Ao

**Affiliations:** 1 Institute of Regulatory Science for Medical Device, National Engineering Research Center for Biomaterials, Sichuan University, Chengdu 610064, China; zhw64@pitt.edu (Z.W.); JIC110@pitt.edu (J.C.); xusong2016@scu.edu.cn (X.S.); 2 Department of Pharmaceutical Science, School of Pharmacy, University of Pittsburgh, Pittsburgh, PA 15261, USA; RUZ33@pitt.edu; 3 Department of Cell Biology, Harvard Medical School, Harvard University, Boston, MA 02115, USA; pearlmoharil@gmail.com; 4 Department of Materials Engineering, Purdue University, West Lafayette, IN 47906, USA; YUL155@pitt.edu; 5 Department of Immunology, School of Medicine, University of Pittsburgh, Pittsburgh, PA 15261, USA; RUNZI@pitt.edu

**Keywords:** polymeric nanoparticles, cancer immunotherapy, micelles

## Abstract

Cancer immunotherapies have generated some miracles in the clinic by orchestrating our immune system to combat cancer cells. However, the safety and efficacy concerns of the systemic delivery of these immunostimulatory agents has limited their application. Nanomedicine-based delivery strategies (e.g., liposomes, polymeric nanoparticles, silico, etc.) play an essential role in improving cancer immunotherapies, either by enhancing the anti-tumor immune response, or reducing their systemic adverse effects. The versatility of working with biocompatible polymers helps these polymeric nanoparticles stand out as a key carrier to improve bioavailability and achieve specific delivery at the site of action. This review provides a summary of the latest advancements in the use of polymeric micelles for cancer immunotherapy, including their application in delivering immunological checkpoint inhibitors, immunostimulatory molecules, engineered T cells, and cancer vaccines.

## 1. Introduction

**Figure 1:**
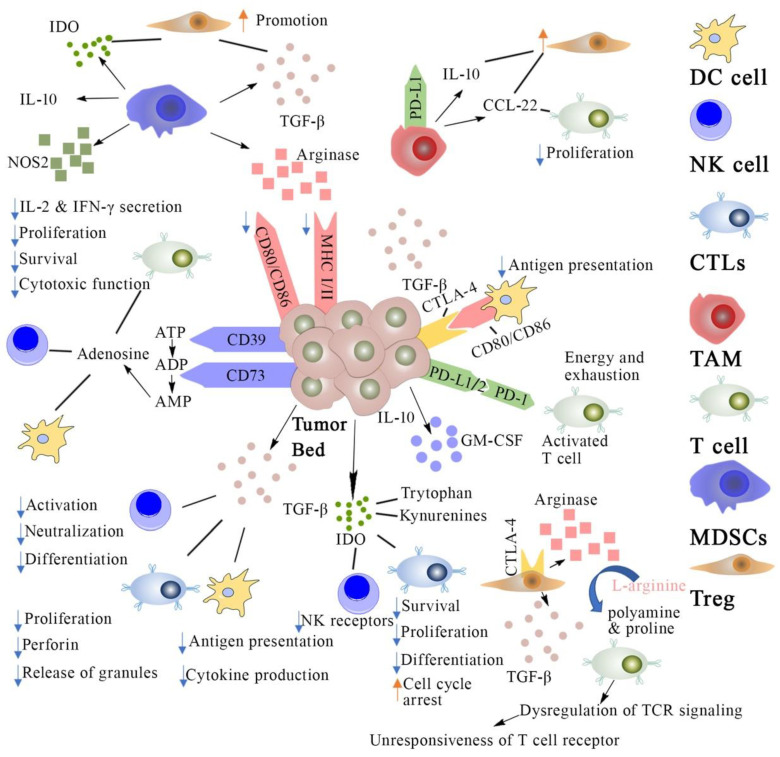
Cancer immunosuppression mechanisms and strategies for cancer immunotherapy. DC cell: dendritic cells; NK cell: natural killer cell; CTLs: cytotoxic T lymphocytes; TAM: Tumor-associated macrophage; MDSCs: myeloid-derived suppressor cells; Treg: regulatory T cell; TCR: T cell receptor; IL: interleukins; PD-L1: programmed death cell ligand 1; PD-1: programmed death cell receptor; MHC: major histocompatibility complex; IDO: indoleamine 2,3-dioxygenase; GM-CSF: granulocyte macrophage colony stimulating factor; ATP: adenosine triphosphate; ADP: adenosine di-phosphate; NOS: nitric oxide synthase; TCF: transforming growth factor.

Cancer immunotherapies have been designed to stimulate our body’s immune system to better recognize and fight cancer [1]. Since bacterial toxins were first reported by William B. Coley for treating sarcoma in patients in 1891, immunotherapies have revolutionized oncology and now offer novel treatment strategies for many types of cancers [2,3]. Cancer immunotherapies aim to “train” our body’s own immune cell population in the tumor microenvironment (TME) for detecting and killing cancerous cells [4,5,6]. There are several types of immune cells that play essential roles in initiating as well as enhancing the anti-tumor immune response such as effector T cells, regulatory T cells, dendritic cells, natural killer (NK) cells, myeloid-derived suppressor cells (MDSCs), and tumor-associated macrophages [3]. Research in the last few decades has shown the development of several strategies for stimulating strong anti-tumor immunity against aberrant cancer cells, such as cancer vaccines, antibodies, and immune-stimulating adjuvants (Figure 1). These strategies are working on re-awakening an immunosuppressive tumor microenvironment as well as enhancing the existing anti-tumor immunity [7].

However, a lot of challenges still remain to be overcome for cancer immunotherapies to be widely applied in the clinical setting, regarding their safety and effectiveness [8]. There are some immunotherapies that are administered as a large dose due to their short half-life, which can result in off-target toxicities and severe side effects (e.g., cytokine release and vascular leakage syndromes) in some patients [9,10,11,12]. Furthermore, only a small proportion of patients respond to cancer immunotherapies (with a 10–30% response rate, depending on the cancer type) in terms of their efficacy [3,13]. It is worthwhile to mention that the majority of current immunotherapies are focused on treating hematological cancers, while only a few of them are approved for treating solid tumors [14]. When we talk of solid tumors, the TME is extremely complex and still not completely understood, and it can pose as a difficult barrier to breakthrough. In addition, while patients with non-immunogenic tumors are reported to respond poorly [15], combination strategy has also demonstrated great potential to strengthen anti-tumor immune response by converting these “cold tumors” to “hot tumors.”

Nanoparticle (NP)-based delivery systems provide a possible solution to the abovementioned challenges. Some nano-sized particles have demonstrated the advantage of preferential accumulation of particles in the tumor owing to the abnormally leaky vasculature and dysfunctional lymphatic system in the TME; a phenomenon termed the enhanced permeability and retention (EPR) effect [16]. In addition, other small-sized NPs have been reported to accumulate in the lymphatic system and enhance the systemic spread of the NP more easily [17]. A variety of nano-based platforms with diverse physicochemical properties have been developed for cancer immunotherapies, including liposomes, polymeric NPs, and silico [8,18,19,20]. NPs made of polymeric micelles have been self-assembled by amphiphilic polymers, consisting of hydrophilic and hydrophobic domains [21,22]. These micelles utilize the core-shell structure for the delivery of small molecules, therapeutic genes, antibodies (Abs), and small interfering RNA (siRNA) [23,24,25]. They stand out among nano-based strategies due to their capability to effectively encapsulate a variety of hydrophobic molecules, high stability in vitro and in vivo, biocompatibility, longevity, and the ability to accumulate in pathological areas with compromised vasculature [26,27,28,29]. In addition, synthetic NPs are easy to chemically modify with additional function to aid the delivery of payloads [30,31,32,33,34]. For example, the surface can be engineered with various ligands and cell-penetrating moieties for specific targeting and intracellular accumulation. Similarly, incorporating stimuli-sensitive functional groups could allow for controlled drug release in response to changes in the certain TME [35,36,37,38].

There is an increasing trend towards designing polymeric micelles by integrating several different functions into a single carrier to create these “smart” multifunctional polymeric micelles [18,23,39]. In this review, we summarized a series of advanced polymeric micelles that have been researched for cancer immunotherapy in order to enhance the efficacy and reduce the side effects associated with the therapy. In addition, we also describe the clinical and preclinical impact of these strategies.

## 2. Main Classification of Cancer Immunotherapies

### 2.1. Cytokines Delivery by Polymeric Micelles

Cytokines are soluble proteins that play an important role in the growth and function of immune cells [40]. In response to a stimulus, cytokines are released and they signal the immune system to exert an immune-modulating effect [41]. In the context of cancer, pro-inflammatory cytokines could contribute to cancer immunotherapy by participating in every phase of the cancer immunity cycle [42,43]. For example, cytokines can improve the antigen priming, increase the number or functionality of immune effector cells in the TME. There are several types of immunostimulatory cytokines, including interferons, interleukins (ILs), tumor necrosis factors, and growth factors. In the clinic, the most commonly used are ILs, interferons, and granulocyte-macrophage colony-stimulating factor (GM-CSF) therapies [44]. When a body gets infected with a pathogen, interferons are produced to induce the activation and maturation of macrophages, lymphocytes, dendritic cells (DCs), and other immune cells [45]. These interferons exhibit strong antiangiogenic activity at the tumor site [46], and the ILs are involved in the activation and differentiation of CD4 T, CD8 T, and B cells to stimulate either the innate or adaptive immune response [47,48,49,50]. There are several common immunostimulatory cytokines under ILs families, such as IL-2 family, IL-10, and IL-12. GM-CSF is a clinically available recombinant cytokine that can promote the expansion, differentiation, and activation of myeloid cells such as DCs and macrophages. In addition, it also contributes to the prime of cytotoxic T lymphocytes (CTLs) [51]. Both granulocyte colony-stimulating factor (G-CSF) and GM-CSF are approved for their application in neutropenia [52]. However, the efficacy of these cytokines is limited by their short half-life and narrow therapeutic window. Therefore, a large dosage is required to achieving good therapeutic efficacy, which causes undesired cytokine release syndrome (CRS) [40,53]. To address the above issues, more actions have been taken, such as designing fine-tuned biomaterials and combining other cytokines, chemotherapies, and immunotherapies to reduce the dose as well as the adverse effects caused by the high dosage (Table 1).

Miki et al. reported an IL-2-encapsulating polymeric micelle by using PEG-polyGlutamate (PEG-pGlu) block copolymer [54]. It has been demonstrated that this system prolonged IL-2 retention in the circulation in comparison to unmodified IL-2 free solution against EG7 tumor-bearing mice. In addition, they also identified that the administration can significantly enhance DC vaccine efficacy against the tumor, along with accumulation of efficient antigen-specific cytotoxic T lymphocytes (CTLs) at the tumor site. Similarly, Liu et al. reported an PMet-P(cdmPEG2K) micellar system, which was based on polymetformin and a chemistry designed to lose the PEG shell in response to the acidic extracellular tumor environment. They demonstrated that this developed PMet-P(cdmPEG2K) micelle co-loaded with Doxorubicin (DOX) and plasmid IL-12 (pIL-12) were more effective at inhibiting tumor growth compared to micelles loaded with DOX or pIL-12 alone [55,56]. Another study aimed to reverse the TAM-mediated immunosuppression in the B16 cancer mouse model by delivering macrophage colony-stimulating factor (M-CSF). Calcium carbonate (CaCO3) was used to crosslink PEG-b-PGA polymers, which would make the micelles sensitive to the acidic extracellular TME. The administration of M-CSF-loaded micelles significantly inhibited tumor growth by enhancing T cell-mediated anti-tumor immune responses [57].

### 2.2. Polymeric Micellar Cancer Vaccines

Vaccines have been proven to prevent some of the deadliest diseases in the twentieth century and have helped save hundreds of millions of lives around the world. There are two types of vaccine therapeutics, including prophylactic vaccines and therapeutic vaccines [58]. In the case of diseases caused by a virus (e.g., polio, smallpox, and measles) and bacteria (e.g., tetanus, tuberculosis, and diphtheria), vaccines achieve their function by exposing people to an inactivated or weakened version of the threat and these vaccines typically work better for the purpose of prevention (given the vaccine before getting infected) [59]. The immune system can identify these threats on the basis of their specific markers, also known as “antigens,” and mount a response against them [60]. However, the situation is far more complicated in the case of a cancer vaccine, which makes it more difficult to develop prophylactic and therapeutic cancer vaccines [61]. Particularly, virus and bacteria appear foreign to our immune system, while cancerous cells have demonstrated many similarities to the healthy cells in our body. Tumor heterogeneity is another major concern because every person’s cancer develops and grows in its own way and is unique in terms of their own distinguishing antigens. As a result, sophisticated and personalized approaches are in urgent need for developing effective cancer vaccines. It is worthwhile to mention that viral infections are responsible for the development of several cancers. Thus, preventative cancer vaccines that target this can play an important role in reducing occurrences. For example, cervical, head and neck cancer can be caused by the human papillomavirus (HPV), while liver cancer can be caused by the hepatitis B virus (HBV). Vaccines have been developed for preventing HPV and HBV infection and reduce the risks of cervical cancers (HPV vaccine) and hepatocellular carcinoma (HBV vaccine) in high-risk populations, and four of them have been approved by the US Food and Drug Administration (FDA), including Cervarix^®^, Gardasil^®^, Gardasil-9^®^, and HEPLISAV-B^®^ [62].

In addition to preventative vaccines, there are four types of therapeutic vaccines under investigation, including DC vaccines, nucleic acids, tumor cell lysates (TCLs), and neoantigens. DCs are majorly responsible for presenting and processing cancer antigens, capable of modulating both innate and adaptive immunity [63,64,65]. DC vaccines are one of the attractive cell-based vaccines due to their low toxicity profile and potential to induce long-term effects via immunological memory. In this process, DCs are obtained from the patients and are stimulated in vitro to express tumor-associated antigens (TAAs) and are then injected back into the patient to directly use the activated T cells for killing cancer cells [66,67]. One DC vaccine, named sipuleucel-T, has been approved by the FDA for prostate cancer treatment, while other DC vaccines have failed in the clinical trials despite their high safety profile. It is predicted that the therapeutic efficacy of DC vaccines can be improved by either increasing the expression level of the target antigen on the surface of the DCs or improving the lymph node delivery efficiency of the DC vaccines [68]. TCLs are activated to contain a variety of TAAs, which can overcome an ineffective immunization in case of a loss of a single antigen after tumor mutation [69]. In general, TCLs are applicable for all kinds of patients and are not restricted by their human leukocyte antigen (HLA) type. Many studies have supported that tumor cells can be modified and engineered to express more immune-associated elements and TCLs can produce a better anti-tumor immunity in vivo post injection. However, a variety of TCL-based vaccines have failed in clinical trial phase II or III. There are a couple of reasons that contributed to this failure. One of them is that there are only a very small proportion of tumor-specific antigens in the whole TCLs, while the whole TCLs have numerous well-characterized and uncharacterized antigens. In addition, TCLs are easily degraded in vivo, and their uptake efficiency by antigen-presenting cells (APCs) is very low [69]. This prompts us to develop new techniques and materials for enhancing its effectiveness [70,71].

The nucleic acid-based vaccine is another promising strategy which works by delivering exogenous nucleic acid into the targeted cells [72]. The nucleic acid therapies include DNAs and RNAs, which can be taken up and translated into antigenic proteins by APCs and induce an effective anti-tumor immune response [73]. DNA-based vaccines have clinically failed due to their limited intranuclear delivery efficiency and disappointingly low immunogenicity [74]. On the other hand, the RNA-based vaccines can be directly translated into antigenic proteins, which leads to enhanced immune efficiency. As a naturally occurring molecule, it is inexpensive and its half-life can be modified easily. More importantly, RNA is not integrated into the genome unlike DNA, and therefore leads to fewer safety concerns. However, the naked RNA is highly susceptible to nuclease degradation and requires proper transfection reagents or delivery techniques so as to enhance the delivery efficiency. Overall, an effective, safe and well-established delivery strategy is highly needed [75,76,77]. Another kind of vaccine named the neoantigen vaccine utilizes tumor somatic DNA as antigen for enhancing anti-tumor immune response [78]. These highly immunogenic neoantigens are specifically expressed in tumor cells and targeting them could avoid damage to normal tissues [79]. They are capable of activating CD4^+^ and CD8^+^ T cells to generate an immune response and have become a new target for cancer immunotherapy [80]. Delivery strategies can improve the stability of these neoantigens and offer the possibilities for co-delivery multiple classes of the vaccine to improve both the safety and efficacy [81,82,83] (Table 2).

It has been widely accepted that lymph nodes (LNs) are critical targets of cancer vaccines because antigen presentation and initiation of T-cell-mediated immune responses occur primarily at these locations. Li et al. developed a mixed micelles system on the basis of amphiphilic diblock copolymer poly(2-ethyl-2-oxazoline)-poly-(d,l-lactide) (PEOz-PLA) and carboxylterminated-Pluronic F127. This system can co-load ovalbumin (OVA) and Toll-like receptor-7 agonist CL264 (carboxylated-NPs/OVA/CL264) to the LN-resident dendritic cells (DCs) [84]. The mixed micelles greatly promoted DC uptake, cytokine production, and the better cross-presentation of antigens to CD8^+^ T cells, leading to the induction of the potent antigen specific cellular immune responses in vivo. In addition, immunization with this codelivery system significantly prevented the E.G7-OVA tumor growth in C57BL/6 mice. Zeng et al. also developed a LN-targeting polymer hybrid micellar system, which are self-assembled from two amphiphilic diblock copolymers, poly-(ethylene glycol) phosphorethanolamine (PEG-PE) and polyethylenimine-stearic acid conjugate (PSA) via hydrophobic and electrostatic interactions [85]. They demonstrated that this novel developed system can encapsulate the melanoma antigen peptide Trp2 and Toll-like receptor-9 (TLR-9) agonist CpG ODN. In addition, the optimal system can potently target proximal LNs where their cargos are efficiently internalized by DCs. In this way, the Trp2/CpG co-delivery system greatly expanded antigen specific cytotoxic T lymphocytes (CTLs) and offered a strong anti-tumor effect in a lung metastatic melanoma model [85]. In addition, improved Trp2 vaccine efficacy was observed in other TME-modulating micelles. The curcumin-PEG micelles reported by Lu et al. led to a significant reduction of myeloid-derived suppressor cells and regulatory T cells (Tregs), decreased levels of IL-6, and chemokine ligand 2, and increased the numbers of CD8^+^ T cells and thereby enhanced the efficacy of Trp2 vaccines [86]. Another strategy enhancing Trp2 vaccine efficacy was reported by Huo et al. The PLGA-PEG micelles loaded with sunitinib base, a tyrosine kinase inhibitor, reduced the number of Tregs in the TME and remodeled vessel density. These led to increased cytotoxic T cell infiltration and enhanced Trp2 vaccine efficacy [87].

Encapsulating antigens in NPs demonstrated distinct advantages in comparison to soluble formulations. For example, this strategy helps to protect antigens from proteolytic degradation and can deliver antigens to DCs in a targeted and prolonged manner. In addition, the delivery system increases the localized dosages into resident immune cells and prevents the antigens from systemic circulation to reduce their toxicity. Most importantly, it significantly enhanced the efficacy in terms of DC presentation, which then recruited cytotoxic T cells (CTLs) and promoted an enhanced anti-tumor immunity. Therefore, a number of NP strategies for delivering antigens have been developed to target DC. Zeng et al. reported that their PSA micelles themselves were a potent adjuvant to enhance the efficacy of the antigen Trp2. In a dose-dependent manner, PSA micelles were able to stimulate bone marrow-derived DC maturation, proliferation, and cytokine secretion. Of note, as protective carriers, the PSA micelles also increased cellular uptake. Compared to free Trp2-PSA mixture delivery, the PSA micelles resulted in significantly higher CTL toxicity and inhibited tumor growth in mice [88]. Rietscher et al. have developed hydrophilic polyethylene-glycol (PEG)-b-PAGE-b-poly (lactic-co-glycolic acid) as a delivery platform for prophylactic vaccination, when encapsulated with ovalbumin (OVA) as a model antigen [89]. In this system, they observed that it significantly enhanced the T cell activation by APCs in comparison to the free soluble OVA antigen.

Targeting DCs offers a promising strategy for improving the efficacy of immunotherapies. There are a number of DC ligands such as fucose, mannose, N-acetyl glucosamine, anti-DEC205, and anti-CD11c. NPs modified with the above ligands have demonstrated a significantly enhanced uptake via receptor-mediated endocytosis in vivo. In addition, the binding of those moieties with their target receptor on the DC can also enhance maturation, which further increases the efficacy of formulated vaccines. Kempf et al. have developed PLGA-NPs with surface-conjugation for targeting DCs. These modified polymeric NPs significantly induce DC maturation as evidenced by the upregulation of DC maturation markers (CD83 and CD86), along with an induction of IL-2 [90].

DNA vaccines in cancer immunotherapy are plasmids delivering genes that encode associated tumor antigens to elicit an adaptive immune response. The SART3 gene encodes a tumor-rejection antigen, a squamous cell carcinoma antigen recognized by T-cells and is under investigation as a novel immunotherapy strategy. Cui et al. and Furugaki et al. reported that polymeric micelles made of P[Asp(DET)]/PEG-b- P[Asp(DET)] prolonged SART3 retention in vivo and observed SART3 protein expression in tumor tissue. The SART3 micelles significantly improved the survival rates of mice bearing CT26 tumors, while delivering naked SART3 genes failed to elicit such therapeutic responses. The elevated activities of CTL and NK cells of splenocytes, as well as the increased infiltration of DCs and CD8a^+^ T cells into tumor tissue, were also observed with the administration of SART3 micelles [91,92]. Polymeric micelles were also used for gene delivery to enhance the efficacy of tumor antigens. PEG-PLL-PLLeu micelles co-delivering STAT3 siRNA and ovalbumin elevated expression levels of CD86 and CD40 as well as IL-12 produced by tumor-associated DCs. These facilitated the maturation and activation of tumor-associated DCs [93,94]. STAT3 is hyperactivated in tumors and associated with less matured tumor-associated DCs, which results in a poor response to TLR stimulation and subsequent poor clinical prognosis [95]. Utilizing siRNA to delete the STAT3 gene in tumor tissue could effectively overcome DC dysfunction and therefore provide a synergistic effect with tumor antigens on eliciting CTL responses [94]. Polymeric micelles are capable of protecting siRNA molecules and antigens while delivering them to the same target sites and therefore ensure the synergistic augmentation of CTL responses.

### 2.3. Immunological Checkpoint Inhibitors Based Polymeric Micelles

Nowadays, it is a well-recognized fact that a cancer cell prevails through alternating the immune microenvironment into their own goods [96]. By expressing immune checkpoint proteins such as cytotoxic T lymphocytes-associated protein-4 (CTLA-4), programmed cell death receptor 1 (PD-1), and programmed cell death ligand-1 (PDL-1), cancer cells can avoid the activation of a T lymphocyte-mediated immune response and survive in the immuno-suppressive niche [97,98]. Thus, Abs were designed to block the inhibition of T-cell function to preserve the anti-cancer activity. The very first FDA-approved checkpoint inhibition immunotherapy employed ipilimumab (Yervoy^®^), a CTLA-4 monoclonal antibody (mAb), to treat melanoma by blocking the CTLA-4 pathway. Currently, multiple T cell-associated inhibitory molecules, such as lymphocytes activation gene-3 (LAG-3, CD223), T cell immunoglobulin-3 (TIM-3), and T cell immunoglobulin and ITIM domain (TIGIT), have been discovered and turned into “druggable” targets [99,100,101]. Apart from the T cell-mediated pathways, non-T cell-related targets such as indoleamine 2,3-dioxygenase (IDO) also play key role in the anti-cancer immune response [102]. The commonly observed overexpression of IDO elevates the level of KYN in tissue milieu, which lowers CD8 T cell activity and creates an immunosuppressive environment. Therefore, IDO inhibitors are considered promising candidates for cancer immunotherapy and a few are under preclinical and clinical exploration, such as indoximod (D-1-MT, NLG-8189), Navoximod (NLG-919), Epacadostat (INCB024360), PI-3065, and PF-06840003 (EOS200271).

As the administration of these immune checkpoint inhibitors increases in patients, their limitations are more understood, such as the immune-related adverse events (irAEs), including thyroid dysfunction and hypophysitis. Additionally, only a small patient population responds to the checkpoint immunotherapy, and a section of them also develops acquired resistance. These limitations make checkpoint blockers work only for a small group of patients while putting them at the risk of numerous complications from the treatment. Polymeric micelles represent a promising strategy to address these issues (Table 3).

In a study conducted by Peng et al., NLG919 was selected as an IDO inhibitor, which targets the tryptophan metabolism, to investigate the enhancement of tumor immunity post photothermal therapy (PTT) [103]. Post PTT, the NLG919/IR780 micelle system could effectively accumulate in the tumor and migrate to lymph nodes, stimulating the activation of T lymphocytes. Chen et al. reported an immunostimulatory and dual functional nanomicellar carrier that is based on a prodrug conjugate of PEG with NLG919. An Fmoc group, an effective drug-interactive motif, is also introduced into the carrier to improve the drug loading capacity and formulation stability. They demonstrated that PEG2k-Fmoc-NLG alone was effective in enhancing T-cell immune responses and exhibited significant anti-tumor activity in vivo. In addition, the systemic delivery of paclitaxel (PTX) using the PEG2k-Fmoc-NLG nanocarrier leads to a significantly improved anti-tumor response in both breast cancer and melanoma mouse models [104]. In the same lab, Sun et al. designed a redox-responsive immunostimulatory polymeric prodrug carrier, PSSN10, for the programmable co-delivery of an immune checkpoint inhibitor NLG and a chemotherapeutic DOX. The PSSN10 carrier dose-dependently enhanced T-cell immune responses in the lymphocyte-Panc02 co-culture experiments, and significantly inhibited tumor growth in vivo. In 4T1.2 tumor-bearing mice, DOX/PSSN10 micelles was more effective than DOXIL (a clinical formulation of liposomal DOX) or free DOX in inhibiting the tumor growth and prolonging the survival of the treated mice. In addition, a more immune-active tumor microenvironment was observed in the mice treated with PSSN10 or DOX/PSSN10 micelles compared with the other treatment group [105].

Moreover, Wan et al. developed an indoximod-based polymeric micelles system for the co-delivery of IDO inhibitor indoximod with Dox, classical chemotherapy for breast cancer. It has been demonstrated that this co-delivery carrier significantly improved the anti-tumor immunity and led to a very strong anti-tumor effect in a preclinical breast cancer model [106]. A novel dual functional indoximod-based carrier PEG2K-Fmoc-1-MT, was developed by Lan et al., for the simultaneous delivery of indoximod and DOX for breast cancer immunochemotherapy. PEG2k-Fmoc-1-MT prodrug micelles presented enhanced inhibition ability of IDO with decreased kynurenine (KYN) production, and an increased proliferation of effector CD4^+^ and CD8^+^ T cells (in a dose-dependent pattern). In combination, DOX triggered an immunogenic cell death action, and the cleaved 1-MT promoted the secretion of tumor necrosis factor-alpha (TNF-α), IL-2 and interferon (IFN)-γ, further facilitating the T cell-mediated immunity. More importantly, the DOX-loaded micelles led to a significantly improved inhibition on tumor growth and prolonged animal survival rate in the 4T1 murine breast cancer model [107]. Huang et al. developed amphiphilic PEG-poly-1-Methyl-l-Tryptophan (MLT) block copolymers, which self-assembled into polymeric micelles in aqueous conditions. The PEG-poly(MLT) block copolymers were able to reduce the levels of KYN in activated macrophages [108].

Substantial research has been done especially intervening in the immune checkpoint between DCs and T cells by utilizing CTLA-4 and PD-1 [109]. In one study, Li et al. developed CTLA-4 small interfering RNA (siRNA) encapsulated poly(ethylene glycol)-block-poly (d,l-lactide) (PEG-PLA) copolymer based nanoparticles, which demonstrated direct and effective T cell activation by targeting immune checkpoint pathways [110]. Copolymer-based nanoparticles have shown to protect siRNA from enzymatic degradation as well as improve its internalization to cells, in comparison to bare siRNAs with the negatively charged surface. These nanocarrier systems might be a potential platform for siRNA delivery targeting the immune checkpoint pathways. Further, as mentioned before, the modification of nanoparticles with specific targeting moieties might enhance the efficiency of antitumor immunity. Su et al. developed a pH and matrix metalloproteinase dual-sensitive micellar nanocarrier, which demonstrated the spatio-temporally controlled release of anti-PD-1 and paclitaxel (PTX) in solid tumors. They showed that antitumor immunity can be activated by PTX-induced immunogenic cell death (ICD), while anti-PD-1 blocked the PD-1/PD-L1 axis to suppress the immune escape due to PTX-induced PD-L1 up-regulation, resulting in a synergistic antitumor immunochemotherapy. Moreover, a sheddable polyethylene glycol (PEG) shell has been decorated, which can mediate the site-specific sequential release of anti-PD-1 and PTX [111].

**Table 3:** Summary of various polymeric micelles systems for checkpoint inhibitors.

Checkpoint Inhibitors	Polymer	Cancer Type	Mechanism of Action	Reference
NLG919	MPEG-PCL	Breast Cancer	Enhance T cell and APC maturation	[103]
NLG919	PEG2k-Fmoc-NLG	Breast Cancer and Melanoma	Enhance T cell	[104]
NLG919	PSSN10	Breast Cancer	Enhance T cell	[105]
Indoximod	POEG-b-PVBIND	Breast Cancer	Enhance T cell	[106]
Indoximod	PEG2k-Fmoc-1-MT	Breast Cancer	Enhance T cell	[107]
1-Methyl-l-Tryptophan	PEG-P(MLT)	Leukemia	APC maturation	[108]
CTLA-4 siRNA	PEG-PLA	Melanoma	Increase proliferation and activity of T cell, depletion of Treg cell	[110]
Anti-PD-1 peptide	Azide-PEG-PAsp (Dip/Bz)	Melanoma	Increase proliferation and activity of T cell	[111]

### 2.4. Molecular Adjuvants

A variety of molecular adjuvants that can stimulate an immune response are under development and have translated into clinic [112,113]. Toll-like receptors (TLRs), expressed on antigen-presenting cells (APCs) such as DCs and macrophages, have been one of the most popular targets [114]. Targeting TLRs can induce a variety of gene expression profiles according to the type of receptors and stimuli, which can stimulate both innate and adaptive immunity [115,116]. TLR-9 is one of the most common receptors, and its ligand is short single-stranded DNA with unmethylated CG motifs, named CpG oligodeoxynucleotides (ODNs) [116]. In general, there are three types of CpG ODNs based on their different biological activities [117]. Some of them have shown great potential for cancer immunotherapy by biasing potent type I helper T (T_h_1) cells. Another common one is TLR-4, and it can be activated by lipopolysaccharides (LPS) [118]. However, LPS demonstrated a high toxic profile, and its derivatives with less toxicity are under exploration. Among them, monophosphoryl lipid A (MPLA) has been developed by removing a phosphate residue, and it demonstrated a 1000-fold decreased toxicity in comparison to LPS. Interestingly, it has already been applied in some clinically explored vaccine formulations. It is worthwhile to mention that despite LPS and MPLA both targeting TLR-4, they have demonstrated different cytokine profiles [119,120,121,122]. Apart from what has been discussed above, other adjuvants targeting other TLR pathways are also actively being explored. For instance, it has been reported that poly (I: C) can activate TLR-3 as a viral RNA mimic.Poly (I: C), a synthetic double-strand RNA, has been investigated as a therapeutic for cancer [123]. It usually complexes with stabilizing molecules such as poly-lysine in order to prevent enzymatic degradation because RNAs are very susceptible in terms of RNase degradation [124]. Molecular adjuvants that activate TLR5 include flagellin, a protein present in bacterial flagella, which can induce the production of tumor necrosis factor-alpha (TNF-r) [125]. In combination with other vaccine antigens, flagella can also significantly improve high antibody titers. Imidazoquinoline derivatives with anti-viral properties can selectively activate TLR-7 and TLR-8. For example, imiquimod can selectively activate TLR-7, and resiquimod (R848) can activate both TLR-7 and TLR-8, which leads to the production of type I interferon and IL-12. It is worthwhile to mention that R837 has been approved by the Food and Drug Administration (FDA) for treating actinic keratosis, basal cell carcinoma, and genital warts [126,127,128,129]. Some other molecular adjuvants targets include stimulator of interferon genes (STING) and nucleotide-binding oligomerization domain (NOD)-like receptors. STING senses cyclic dinucleotides and nucleic acids of bacterial and viral origin. STING pathway activation leads to type I IFN secretion upon infection. Cyclic di-AMP and cyclic-dimeric guanosine monophosphate (GMP) are cyclic dinucleotides originating from bacteria that have been used as STING agonists for vaccine development. These cyclic dinucleotides can induce the production of cytokine-mediated by type I IFN and NF-kB pathways. NOD-like receptors can regulate inflammation and innate immunity by inflammasomes. The synthetic adjuvants molecules such as muramyl dipeptide can activate NOD2, which can lead to the production of pro-inflammatory cytokines such as IL-1, IL-6, IL-8, and TNF-alpha [130,131,132,133,134]. In addition to the above STING modulators, there are several other STING agonists such as ADU-S100 (MIW815), MK-1454, CGAMP, and MSA-2 [135].

Adjuvants are known to enhance immune responses by activating antigen-presenting cells and facilitating antigen presentation. Presenting both antigens and adjuvants to the same antigen-presenting cells is crucial for eliciting sufficient pharmacological responses for cancer immunotherapy [136]. Therefore, co-delivering antigens and adjuvants will provide better therapeutic outcomes. However, many adjuvants are nucleic acids, peptides, and nuclei proteins that are known for their instability in vivo. Adjuvant degradation before reaching the target cells and low abundance in target sites hamper the efficacy of current cancer immunotherapy. To improve the efficacy, many drug delivery strategies using polymeric micelles as platforms to co-deliver antigens and adjuvants are under investigation. Polymeric micelles can not only prolong adjuvants retention in lymphoid organs but also increase adjuvant accumulation in tumors (Table 4).

Tumor-associated macrophages (TAMs) are usually polarized to an activated M2 phenotype, which exerts numerous tumor-promoting features, such as the enhancement of angiogenesis and the suppression of the immune response [137]. TAM-targeting immunotherapy is a greatly promising strategy that involves altering the TME with the immunomodulator imiquimod (R837) for enhanced cancer therapy. However, the function of R837 is seriously limited by its poor water solubility and a lack of targeting ability. Wei et al. developed two types of targeting polymeric micelles for combination chemo-immunotherapy against breast cancer. One micelle (ACP-R837) is immunostimulatory, which is formed by the self-assembly of the AC-CS-PpIX (ACP) polymer, which contains acetylated-chondroitin sulfate (AC-CS) as the hydrophilic block and protoporphyrin IX (PpIX) grafted onto AC-CS as the hydrophobic block [138]. ACP-based micelle has the capacity to selectively deliver R837 to TAMs due to the affinity of CS (the major component of ACP) for the mannose receptor (CD206). The other micelle is with chemotherapeutic function (PPP-DOX), which is formed from a phenylboronic acid (PBA) functionalized polyethylene glycol (PEG)-polycaprolactone (PCL) copolymer (PBA-PEG-PCL). This micelle can deliver the chemotherapeutic drug DOX to breast cancer cells via receptor-mediated endocytosis. Through the combined delivery of the two types of micelles, the chemotherapeutic micelle actively targeted tumor cells while the immune-stimulatory micelle selectively targeted TAMs. The immune-based micelle stimulated macrophage maturation. Upon the activation of the macrophages, the inhibitory effect of the chemo-based micelle against tumor cells was markedly increased. Overall, the combined polymer micelles significantly activated the anti-tumor immune response within the tumor microenvironment and achieved an excellent in vivo antitumor effect. Li et al. and Ni et al. reported that polymeric micelles made from PCL-PEI/PCL-PEG and PEG-PLA could increase antigen/adjuvant uptake by DCs and prolong their retention in the lymph nodes [139,140]. Besides, Cui et al., Furugaki et al., and Coumes et al. reported that antigens and adjuvants co-delivered via polymeric micelles successfully inhibited tumor growth and metastasis in mice as well as prolonged tumor-bearing mice survival, compared to free antigen–adjuvant combinations [91,92,141,142]. The improved pharmacological responses are also supported by the observations of increased CD11c^+^ DCs and CD4^+^/CD8a^+^ T cells infiltration into tumors, stronger CTL activities, and higher CD4^+^IFN-ɣ (Th1) response [92,139,143].

In addition to being protective carriers, polymeric micelles can also facilitate in eliciting immune responses with the adjuvants. Liu et al. had reported that their PEG-PE micelles served as a chaperone for the adjuvant, MPLA, in TLR signaling to activate DCs. Moreover, the PEG-PE micelles could assist protein folding, and therefore, these micelles converted the antigen peptides into α-helix structures, which avoided antigen aggregation and achieved sufficient cystolic antigen delivery. In mice bearing TC-1 tumors, these micelles had increased the cross-priming of CD8^+^ T cells and induced the memory of CTLs. They further reported that combining these micelle vaccines with chemotherapy or surgical operation led to reduced tumor relapse and resulted in long-term protective immunity in mice [144].

Moreover, encapsulating immunomodulatory drugs in micelles can enhance physiological properties as well as targeting the ability of such molecules.

Vinod et al. developed poly(2-oxazoline) (POx)-based nano-micellar formulation of R848, a TLR 7/8 agonist, and characterized its function in a clinically relevant mouse model of metastatic non-small cell lung cancer (NSCLC). POx is an amphiphilic triblock copolymer composed of one hydrophobic block of poly(2-butyl-2- oxazoline) (BuOx) flanked by two hydrophilic blocks of (2-methyl-2-oxazoline) (MeOx). POx micelles exhibit an exceptionally high solubilization capacity for water-insoluble drugs in either a single drug- or multidrug-loaded setting [145,146]. They have shown that this POx-R848 formulation demonstrated a superior tumor-inhibiting effect in a metastatic model of NSCLC, relative to anti–PD-1 therapy or platinum-based chemotherapy. Investigation of the in vivo immune status showed that the R848-based stimulation of antigen-presenting cells in the tumor microenvironment resulted in the mobilization of an antitumor CD8^+^ immune response [147].

**Table 4:** Summary of various polymeric micelles systems for molecular adjuvants.

Adjuvants	Polymer	Cancer Type	Mechanism of Action	Reference
R837	AC-CS-PpIX (ACP) polymer	Breast Cancer	Targeting TAMs	[138]
	PBA-PEG-PC			
R848 CpG	PEG-PLA	Colon Cancer	TLR 7/8 and TLR 9 agonist	[139,140]
SART3/CD40L/GM-CSF pDNA	P[Asp(DET)] PEG-P[Asp(DET)]	Colon Cancer		[91]
MPLA	PEG-PE	Lung Cancer	TLR signaling to activate DCs	[144].
R848	POx-R848	NSCLC	Th1 polarization	[147]

### 2.5. Modulation on TME Using Polymeric Micelles

Altering the immunosuppressive TME can also be achieved with the use of other immunomodulatory polymer or drugs [148]. A self-assembled cationic amphiphilic polymeric micelle system was developed by Hyeona Yim et al., composed of a hydrophobic ATRA (all-trans-retinoic acid), a hydrophilic low molecular PEI, and the shielding agent hyaluronic acid. This system had a strong positive charge (~ +40 mV), which resulted in mechanical disruption and necrosis in cancer cells in vitro. Furthermore, the organelle fragments induced by this necrotic cell death could produce a large amount of cell debris, giving rise to neoantigens sufficient to trigger a strong antitumoral immune response and synergistically reduce tumor burden in vivo. This event is also known as the “abscopal effect.” However, this event is short-lived and weak, and studies have shown that the additional administration of NPs can specifically absorb these neoantigens and enhance the efficacy of antigen-presenting cells (APCs) in the TME [149].

The TME possesses several abnormal conditions, including hypoxia, immunosuppression, acidic pH, increased vessel density, and condensed extracellular matrix (ECM), around the tumor tissue. The TME is related to drug resistance and modulating the TME can improve treatment efficacy without increasing drug doses. Administrating pre-treatments to modulate the TME before cancer therapy has shown improved efficacy. For example, pre-treatment with ambroxol, hyaluronidase, and imatinib mesylate could enhance micelle distribution and accumulation at tumor sites by normalizing tumor vessels and reducing vessel density [150,151,152]. Polymeric micelles are versatile platforms that can easily incorporate TME-modulating compounds. Thus, modulating the TME using polymeric micelles may produce better outcomes for cancer immunotherapy. Reversing angiogenesis has also been reported to improve the outcomes of cancer immunotherapy. Benny et al. reported their PEG-PLA micelles loaded with lodamin, an oral nontoxic antiangiogenic compound, led to reduced tumor cell proliferation and enhanced tumor apoptosis. These micelles also prevented the development of liver metastasis from B16/F10 tumor cells injected into mouse spleens [153]. In addition, glycolipid micelles that incorporate telmisartan selectively eliminated the stromal cancer-associated fibroblasts and reversed drug resistance caused by the fibroblast secreted cytokines [154]. Moreover, Wang et al. utilized PEG-PAA-PS micelles loaded with zinc phthalocyanine, a compound with oxygen self-compensating ability, in photodynamic therapy to generate more singlet oxygen. This reversed the hypoxic TME and resulted in higher photocytotoxicity [155].

### 2.6. Engineered T Cells

Engineered T cells consist of chimeric antigen receptor (CAR)-T cells and T cell receptor cells (TCR T cells). In general, CAR-T cells are autologous T cells derived from the peripheral blood of patients, which have been engineered in vitro and transfused back in vivo. These modified T cells express CARs that specifically recognize tumor antigens and can recognize and kill tumors in an efficient way [156,157]. In addition, these T cells maintain their activity for a long time in the body. To date, there are several clinical trials for CAR-T cells undergoing, and the majority of them are focusing on B-cell malignancies. In 2017, two CD19 targeting therapies were approved for lymphoma. CD19 is primarily expressed in B cell leukemia and lymphoma, while normal B cell lineages also express CD19 molecules [9]. The main side effect of CD19 targeting CAR T therapy is B cell hypoplasia, which can be alleviated by immunoglobulin replacement therapy. Although there are many encouraging results in the clinic, CAR-T therapy still has a lot of challenges to be overcome. One of the major concerns about CAR-T therapy is its long-term safety profile. CAR-T cell- based therapy causes two life-threatening toxicities: CRS and neurotoxicities. In addition, the therapeutic outcome of CAR-T is inversely affected in terms of treating solid tumors with a harsh TME [158,159,160,161,162,163,164,165,166,167].

TCR-T cell therapy is an MHC-dependent immunotherapy. TCRs respond to MHC-presented tumor-associated intracellular antigens, such as cancer-testis antigens and neoantigens [168,169,170]. Currently, TCR T cell therapies are available both for hematological and solid tumors. Overall, novel biomaterials for the improvement of the therapeutic efficacy as well as the reduction of their adverse side effects are urgently needed for CAR-T therapies.

To date, there are few groups that are investigating polymeric micelle systems for engineered T cells. Fan et al. reported a three-segment amphiphilic co-polymer, methoxy polyethylene glycol-branched polyethyleneimine-poly (2-ethylbutyl phospholane) (mPEG-bPEI-PEBP), which was capable of delivering the CAR and packaging plasmids to co-transfect Jurkat cells and undergo expression. They demonstrated that the obtained CAR-Jurkat cells had the ability to secrete interferon-γ and interleukin-2. The cytotoxic effects to the CD19-K562 cells suggest that the induced CAR-Jurkat cells have an excellent targeted antitumor activity [171].

## 3. Conclusions and Future Perspective

In this review, we have summarized different polymeric micelles nano-strategies for improved cancer immunotherapy, including delivering antigens, Abs, cytokines, immunostimulatory molecules such as adjuvants, and immune checkpoint inhibitors. In addition, we have described that these nanocarriers can improve the therapeutic efficacy and reduce the adverse effects of their delivered model drug. It is hoped that the strategies described in this review can be innovatively translated for cancer immunotherapy, and thus can open a new door for a nano-based immunotherapy era.

Since their invention, polymeric micelles have come a long way and scientists have repeatedly attempted to expand their applications in cancer research. Polymeric micelles can pose as an ideal drug delivery system given their numerous advantages—improved half-life, controlled biodistribution and targeting [172], as well as the ability to deliver multiple therapeutics to the same site—which can overcome several translational barriers [173]. When patients are on combination therapy, different chemo- or immune-therapies are dosed via various routes of administration, each with a distinct pharmacokinetic and biodistribution profile [174]. Ideally, if we can deliver such therapies in a single delivery system as a “magic bullet”, we can ensure that the tumor microenvironment is exposed to the combination at the same time, with hopefully the best outcome for patients. Engineering and developing the structure of these polymeric micelles is also a hot topic of research for controlled drug release [39]. This chemical flexibility of the core/shell structure can be tuned to prepare stable and stimuli-sensitive formulations. Some the successful approaches to control drug release include the attachment of hydrophobic structures [175], the introduction of drug compatible moieties, chemical core crosslinking and partial crystallization of the micellar core [176].

As researchers continue to understand the TME and its vulnerabilities, more and more immunotherapies are entering clinical trials. The progress in the field of delivery systems has expanded to smartly deliver various types of therapies successfully, and it is only a matter of time before many other micellar formulations enter the stage of clinical evaluations. With a vast number of polymers approved by the FDA, the advent of polymeric micelles to clinical stages for immunotherapy is expanding and appears to be promising in the near future.

## Figures and Tables

**Table 1:** Summary of various polymeric micelles systems for cytokine delivery.

Cytokines	Polymer	Cancer Type	Mechanism of Action	Reference
IL-2	PEG-pGlu block copolymer	Lymphoma	Enhance DC vaccine	[54]
IL-2 Plasmid	PMet-P(cdmPEG2K)	Breast Cancer	Enhance T cell response	[55]
IL-2 Plasmid	HA-PMet	Breast Caner	Enhance T cell response	[56]
M-CSF	PEG-b-PGA	Melanoma	Enhance T cell response	[57]

**Table 2:** Summary of various polymeric micelles systems for cancer vaccination. LNs: lymph nodes.

Polymer	Mechanism of Action	Adjuvant and Immunogen	Cancer Type	Reference
PEOz-PLA and carboxylated-Pluronic F127	LNs targeting	Ova and CL264	Lymphoma	[84]
PEG-PE and PSA	LNs targeting	Trp2 and CpG	Metastatic melanoma	[85]
Curcumin-PEG	Reduction of MDSCs and Tregs and increased CD8 T cells	Trp2	Melanoma	[86]
PLGA-PEG		Trp2	Melanoma	[87]
PSA	DC targeting	Trp2	Melanoma	[88]
PEG-b-PAGE-b-PLGA	-	Ova	-	[89]
PLGA-NPs	DC targeting	CD40, Fcg, avb3 and avb5 integrin receptors antibodies	-	[90]
P[Asp(DET)]/PEG-b- P[Asp(DET)]	Elevated CTLs and NK	SART3	Colon cancer	[91,92]
PEG-PLL-PLLeu	DC activation	STAT3 siRNA and Ova	Melanoma	[93,94]

## Data Availability

Not applicable.

## Abbreviations

AMP	adenosine monophosphate
APC	antigen presenting cells
ATRA	all-trans-retinoic acid
CAR	Chimeric antigen receptor
CRS	cytokine release syndrome
CSF	colony stimulating factor
CTL	cytotoxic T lymphocytes
CTLA-4	cytotoxic T lymphocytes-associated mocleture-4
DOX	Doxorubicin
DC	dendritic cells
DNA	deoxyribonucleic acid
ECM	extracellular matrix
EPR	enhanced permeability and retention
FDA	United States Food and Drug Administration
G-CSF	granulocyte colony stimulating factor
GM-CSF	granulocyte macrophage colony stimulating factor
GMP	dimeric guanosine monophosphate
HBV	hepatitis B virus
HLA	human leukocyte antig
HPV	human papilloma virus
IDO	indoleamine 2,3-dioxygenase
IFN	interferons
IL	interleukins
ITIM	immunoreceptor tyrosine-based inhibitory motif
LAG-3	lymphocytes activation gene-3
LIF	leukemia inhibitory factor
LPS	lipopolysaccharides
MHC	major histocompatibility complex
MPLA	monophosphoryl lipid A
NF-kB	Nuclear Factor kappa-light-chain-enhancer of activated B cells
NK	natural killer Cells
NOD	nucleotide-binding oligomerization domain
NOD2	nucleotide-binding oligomerization domain-containing protein 2
NP	nanoparticle
OVA	ovalbumin
P[Asp(DET)]/PEG-b-P[Asp(DET)]	polyethylene glycol-b-poly{N’-[N-(2-aminoethyl)-2-aminoethyl] aspartamide}
PCL-PEG	poly(caprolactone)–poly(ethylene glycol)
PCL-PEI	poly(caprolactone)-polyethyleneimine
PD-1	programmed cell death receptor 1
PDL-1	programmed cell death ligand-1
PE	phosphatidylethanolamine
PEG	polyethyleneglycol
PS	polystyrene
PEG-PLL-PLLeu	poly(ethylene glycol)-b-poly(l-lysine)-b-poly(l-leucine)
PEI	polyethylenimine
PGA	polyglutamic acid
PLA	polylactic acid
PLGA-NPs	poly(lactic acid-co-glycolic acid)-nanoparticles
PLGA-PEG	poly(lactic acid-co-glycolic acid)-poly(ethylene glycol)
PSA	polyethylenimine (2k)-stearic acid
PTT	photothermal therapy
RNA	ribonucleic acid
STING	stimulator of interferon gens
TAM	tumor-associated macrophages
TC-1	tissue culture number one
TCR	T cell receptor
TIGIT	T cell immunoglobulin and ITIM domain
TIM-3	T cell immunoglobulin-3
TLR	toll-like receptors
TME	tumor microenvironment
TNF-r	tumor necrosis factor-alpha
